# Antibiofilm Activities of Halogenated Pyrimidines Against Enterohemorrhagic *Escherichia coli* O157:H7

**DOI:** 10.3390/ijms26031386

**Published:** 2025-02-06

**Authors:** Hyejin Jeon, Yong-Guy Kim, Jin-Hyung Lee, Jintae Lee

**Affiliations:** School of Chemical Engineering, Yeungnam University, Gyeongsan 38541, Republic of Korea; hyejin7882@ynu.ac.kr (H.J.); yongguy7@ynu.ac.kr (Y.-G.K.)

**Keywords:** antibiofilm, antimicrobials, curli, *Escherichia coli*, halogenated pyrimidines

## Abstract

Enterohemorrhagic *Escherichia coli* (EHEC) is a significant public health concern due to its ability to form biofilms, enhancing its resistance to antimicrobials and contributing to its persistence in food processing environments. Traditional antibiotics often fail to target these biofilms effectively, leading to increased bacterial resistance. This study aims to explore the efficacy of novel antibiofilm agents, specifically halogenated pyrimidine derivatives, against EHEC. We screened pyrimidine and 31 halogenated pyrimidine derivatives for their antimicrobial and antibiofilm activities against EHEC using biofilm quantification assays, SEM analysis, motility, and curli production assessments. Our findings reveal that certain halogenated pyrimidine derivatives, notably 2-amino-5-bromopyrimidine (2A5BP), 2-amino-4-chloropyrrolo[2,3-d]pyrimidine (2A4CPP), and 2,4-dichloro-5-iodo-7H-pyrrolo[2,3-d]pyrimidine (2,4DC5IPP) at 50 µg/mL, exhibited significant inhibitory effects on EHEC biofilm formation without affecting bacterial growth, suggesting a targeted antibiofilm action. These compounds effectively reduced curli production and EHEC motility, essential factors for biofilm integrity and development. qRT-PCR analysis revealed that two active compounds downregulated the expression of key curli genes (*csgA* and *csgB*), leading to reduced bacterial adhesion and biofilm formation. Additionally, in silico ADME–Tox profiles indicated that these compounds exhibit favorable drug-like properties and lower toxicity compared with traditional pyrimidine. This study highlights the potential of halogenated pyrimidine derivatives as effective antibiofilm agents against EHEC, offering a promising strategy for enhancing food safety and controlling EHEC infections. The distinct mechanisms of action of these compounds, particularly in inhibiting biofilm formation and virulence factors without promoting bacterial resistance, underscore their therapeutic potential.

## 1. Introduction

Enterohemorrhagic *Escherichia coli* O157:H7 (EHEC) is a significant pathogen responsible for severe foodborne illnesses globally. Characterized by its low infectious dose and severe outcomes, such as hemorrhagic colitis and hemolytic uremic syndrome, EHEC presents substantial challenges to public health systems [1]. One of the formidable aspects of EHEC is its ability to form biofilms on various surfaces, which enhances its resistance to conventional antimicrobial treatments and facilitates its persistence in food processing environments [2]. Biofilms are complex communities of bacteria that are embedded in a self-produced extracellular matrix, adhering to each other and to surfaces. The formation of EHEC biofilms is facilitated by the expression of various virulence factors, including curli fibers and other adhesive fimbriae, which are critical for biofilm structural integrity and bacterial adherence to surfaces [3]. This biofilm matrix not only protects EHEC from environmental stresses but also from antimicrobial agents, thereby enhancing the pathogen’s survival and virulence [4]. Consequently, biofilm control is a promising strategy to combat EHEC infections, as it targets the protective barrier and virulence mechanisms of the bacteria. In response to the limitations of traditional antibiotics, which often fail to effectively target biofilm-associated bacteria and often lead to increased resistance, the current study explores the efficacy of novel antibiofilm agents, specifically halogenated pyrimidine derivatives.

Preliminary findings have shown that several halogenated compounds have demonstrated antimicrobial and antibiofilm effects on EHEC. For example, 5-iodoindole [5] and bromoindoles [6] inhibited EHEC formation, and chloroindoles inhibited toxin production and biofilm formation and toxin production by uropathogenic *E. coli* [7]. Recently, it was proposed that halogenated compounds would improve, target binding affinity for enhanced antimicrobial activities, and reduce their toxicity [8]. Heterocyclic pyrimidine scaffolds have demonstrated various biological activities, including antioxidant, antimicrobial, antiviral, anti-inflammatory, and anticancer effects [9]. Additionally, the pyrimidine core is a versatile structure that allows for easy functionalization, making it advantageous for identifying antibacterial agents. Notably, the presence of an ${NH}_{2}$ (amino group) at the C-2 position of the pyrimidine nucleus has been reported to significantly enhance antibacterial activity against pathogenic Gram-positive and Gram-negative bacteria [10]. The selection of halogenated pyrimidine compounds was based on their biochemically stable structures and their ability to interact with nucleic acids, proteins, and metabolites [11]. Previous studies have demonstrated the substantial pharmacological potential of pyrimidine-containing hybrid compounds, identifying them as promising candidates for further investigation [12]. In particular, 5-halopyrimidines, a subclass of C(5)-modified nucleosides, have been reported to possess anticancer, antiviral, radiosensitizing, and photosensitizing properties [13]. More recently, studies have been conducted using halogenated pyrimidines against *Staphylococcus aureus* [14] and *Vibrio parahaemolyticus* [15]. These studies have demonstrated the remarkable antimicrobial properties and biofilm-inhibitory effects of halogenated pyrimidine compounds, providing valuable references for designing diverse substituents.

Hence, this study explored the antimicrobial and antibiofilm potential of various halogenated pyrimidine analogs, particularly having indole-like moiety, against EHEC and found three halogenated aminopyrimidines. To understand the mechanistic insights of the hits, killing dynamics, microscopic observation, curli and motility assays, transcriptomic analysis, and in silico and in vivo toxicity evaluation were performed.

## 2. Results

### 2.1. Antibiofilm and Antimicrobial Effects of Halogenated Pyrimidines Against EHEC

**Figure 1:**
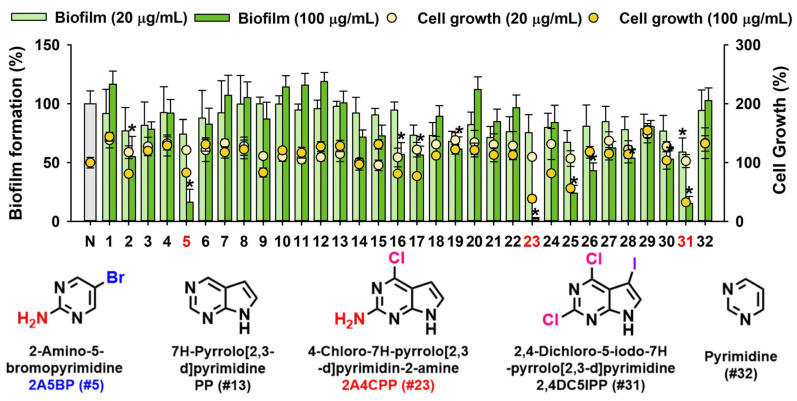
Antibiofilm screening of pyrimidine and 31 halogenated pyrimidine derivatives and pyrimidine against EHEC. Biofilm formation (indicated as bars) was quantified after 24 h culture in 96-well plates without shaking in the presence of each compound at 0, 20, or 100 μg/mL. Planktonic cell growth was also measured and indicated as dots. The red-fonted numbers 5, 23, and 31 in the x-axis of the upper panel represent the most active compounds. * *p* < 0.05 versus non-treated controls. “N” indicates “none”, referring to the absence of any treatment. Full chemical names and structures are shown in Table S1.

The antibiofilm activities of pyrimidine and 31 halogenated pyrimidine derivatives on EHEC biofilms were initially screened using 96-well plates at concentrations of 20 and 100 µg/mL. Overall, the halogenated pyrimidine derivatives displayed varying degrees of inhibitory activity against the formation of EHEC biofilms (Figure 1). Detailed results with the chemical structures of these compounds are provided in Table S1. Notably, 2-amino-5-bromopyrimidine (#5, referred to as 2A5BP), 2-amino-4-chloropyrrolo[2,3-d]pyrimidine (#23, referred to as 2A4CPP), and 2,4-dichloro-5-iodo-7H-pyrrolo[2,3-d]pyrimidine (#31, referred to as 2,4DC5IPP) exhibited significant antibiofilm activity. In contrast, pyrimidine (#32) showed no such effect (Figure 1). Specifically, at a concentration of 100 µg/mL, 2A5BP, 2A4CPP, and 2,4DC5IPP reduced EHEC biofilm formation by more than 50%. Chemically, 2A5BP and 2A4CPP both feature an amine group paired with a halogen atom, whereas 2,4DC5IPP contains two chlorine atoms and one iodine atom.

**Figure 2:**
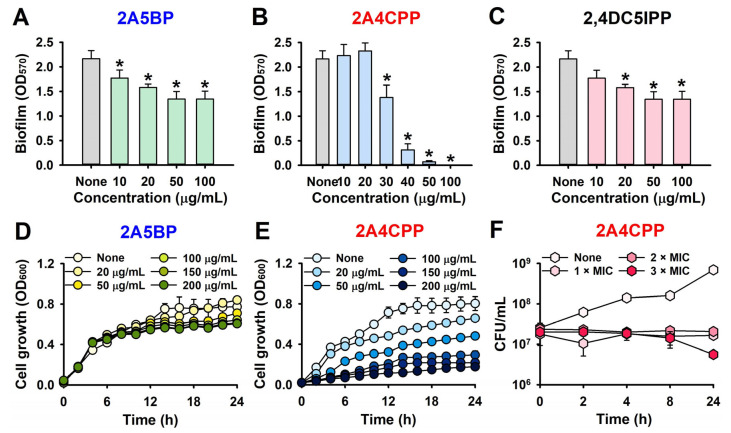
Effects of halogenated pyrimidines on biofilm formation and cell growth: EHEC biofilm formation was quantified with or without the presence of 2A5BP (**A**), 2A4CPP (**B**), or (2,4DC5IPP) (**C**) after 24 h culture in 96-well polystyrene plates. Planktonic cell growths in the presence of 2A5BP (**D**) and 2A4CPP (**E**). Bacteriostatic activity of 2A4CPP (**F**). * *p* < 0.05 versus non-treated controls.

A more detailed biofilm assay revealed that the three compounds dose-dependently inhibited EHEC biofilm formation (Figure 2A–C). For instance, 2A4CPP, when used at concentrations above 50 µg/mL, inhibited EHEC biofilm formation by more than 95%. Consequently, 2A5BP and 2A4CPP were selected for further analysis based on their potent antibiofilm activities.

The effects of halogenated pyrimidine derivatives on planktonic cell growth were assessed at concentrations of 20 and 100 µg/mL over a 24 h culture period (Figure 1). Additional growth curves were generated for 2A5BP and 2A4CPP. 2A5BP did not significantly affect planktonic cell growth up to 200 µg/mL, whereas 2A4CPP exhibited a dose-dependent inhibition of EHEC planktonic cell growth (Figure 2D,E). The minimum inhibitory concentration (MIC) for 2A5BP was determined to be above 600 µg/mL, while for 2A4CPP, it was 400 µg/mL.

To investigate whether the effect of 2A4CPP was bactericidal or bacteriostatic, higher doses were tested. Colony-forming unit (CFU) counting assays revealed that 2A4CPP at 1×MIC did not reduce CFU numbers after 24 h of culture, confirming its bacteriostatic activity (Figure 2F). These results suggest that the antibiofilm activity of 2A5BP does not stem from cell growth inhibition, while the antibiofilm effect of 2A4CPP is largely attributable to its inhibitory impact on cell growth. Consequently, it appears that 2A5BP and 2A4CPP may exert their effects through different mechanisms beyond mere antibacterial activity.

### 2.2. Effect of Halogenated Pyrimidines on Motility and Curli Production

**Figure 3:**
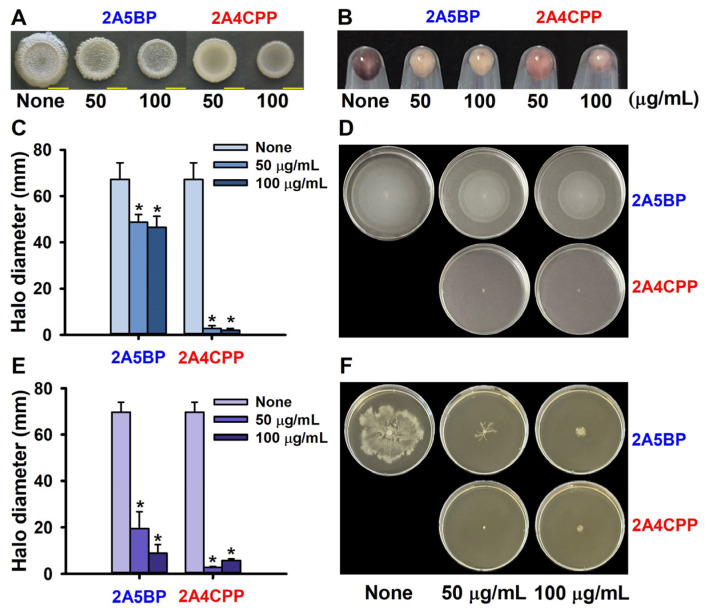
Effects of halogenated pyrimidines on curli production and cell motility: Colony morphology in the presence of 2A5BP and 2A4CPP (**A**). Curli formation in the presence of 2A5BP and 2A4CPP with Congo red staining (**B**). Swimming motility in the presence of 2A5BP and 2A4CPP (**C**,**D**). Swarming motility (**E**,**F**). * *p* < 0.05 versus non-treated controls. Yellow scale bars represent 0.5 cm (**A**).

The effects of 2A5BP and 2A4CPP on cell morphology were observed initially, revealing that treatment with 2A5BP resulted in relatively rough cell morphology, while treatment with 2A4CPP led to comparatively smooth cell morphology (Figure 3A).

Given the pivotal role of *E. coli* cell motility in biofilm formation [16] and pathogenesis [17], the impact of the two active compounds on swimming and swarming motility was examined. 2A5BP, at concentrations up to 100 µg/mL, slightly inhibited swimming motility, whereas 2A4CPP demonstrated significant motility inhibition starting at 50 µg/mL (Figure 3C,D). Quantitatively, 2A5BP at 100 µg/mL resulted in a 35% reduction in motility, and 2A4CPP at 50 µg/mL achieved more than a 95% reduction. The inhibition of swarming motility by two compounds was more significant (Figure 3E,F). These results suggest that the antibiofilm activity of 2A5BP is partially attributable to its ability to inhibit motility, while the predominant antibiofilm mechanism of 2A4CPP is related to its substantial inhibition of motility.

Since EHEC curli fimbriae play a crucial role in its attachment and biofilm formation [18,19], the impact of two active compounds, 2A5BP and 2A4CPP, on curli production was explored. The assessment was conducted using Congo red dye and further analyzed by scanning electron microscopy (SEM). Interestingly, 2A5BP, at concentrations above 50 µg/mL, significantly inhibited curli production, whereas 2A4CPP at the same concentration showed a less pronounced effect on curli formation (Figure 3B). These findings suggest that the primary mechanism of antibiofilm activity for 2A5BP involves the inhibition of curli fimbriae production.

### 2.3. Microscopic Observations of EHEC Biofilm and Curli

**Figure 4:**
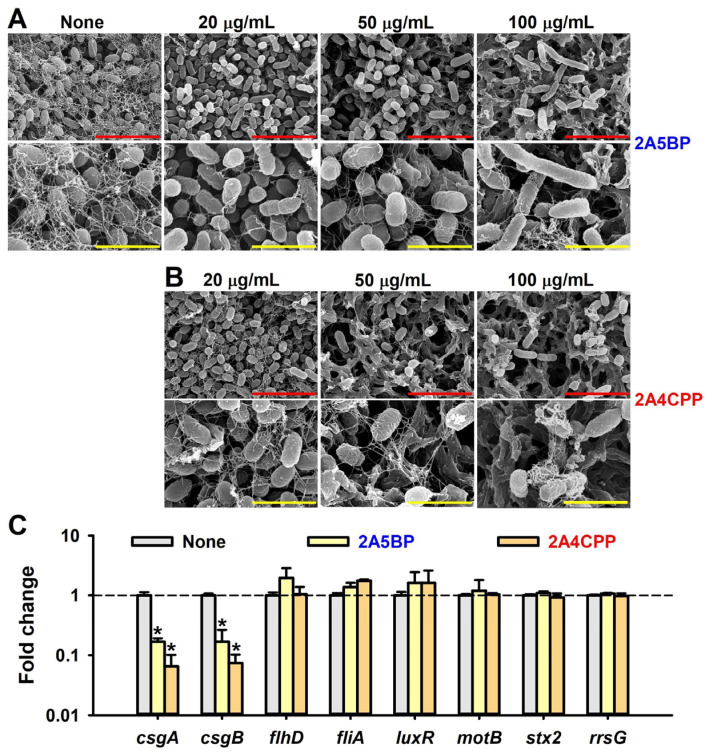
SEM analysis of EHEC biofilms and qRT-PCR analysis. SEM images of cells treated with 2A5BP (**A**) or 2A4CPP (**B**) and gene expression by 2A5BP and 2A4CPP (**C**). Red and yellow scale bars represent 5 µm and 2 µm, respectively. * *p* < 0.05 versus non-treated controls.

SEM was also utilized to visualize the structural effects of halogenated pyrimidines on EHEC biofilms and curli production. The untreated control displayed a robust biofilm structure with numerous curli fibers interconnecting the cells. In contrast, treatment with 2A5BP and 2A4CPP resulted in a dose-dependent reduction in biofilm cell density (Figure 4A,B). Notably, a concentration of 20 µg/mL of 2A5BP completely abolished curli production, while 2A4CPP at 100 µg/mL significantly reduced it. These observations are consistent with the results from the Congo red curli assay. Interestingly, SEM analysis also revealed that treatment with 2A5BP led to elongated cell sizes. Specifically, while the average cell size in the untreated control was 1.32 ± 0.26 µm, cells treated with 2A5BP at 100 µg/mL expanded to an average of 2.47 ± 1.79 µm. This suggests that 2A5BP may inhibit cell division, contributing further to its antibiofilm activity.

### 2.4. Differential Gene Expression Analysis of EHEC Induced by 2A5BP and 2A4CPP

To investigate the antibiofilm mechanisms of 2A5BP and 2A4CPP in EHEC, the expression levels of eight biofilm-related genes were analyzed using real-time qRT-PCR. 2A5BP and 2A4CPP had no effect on the expression of the housekeeping gene (*rrsG*) (Figure 4C). However, they significantly inhibited the expression of *csgA* and *csgB*, which regulate curli production, leading to reduced bacterial adhesion and biofilm formation. Specifically, the gene expression of *csgA* showed a 6-fold reduction with 2A5BP and a 15-fold reduction with 2A4CPP, while *csgB* exhibited a 6-fold reduction with 2A5BP and a 13-fold reduction with 2A4CPP. In contrast, no significant changes were observed in the expression levels of genes related to flagellar formation and motility (*flhD*, *fliA*, *motB*), quorum sensing (*luxR*), and Shiga toxin production (*stx2*).

### 2.5. ADME–Tox Profiling of Halogenated Pyrimidines

In silico ADME–Tox profiling revealed that both 2A5BP and 2A4CPP conformed to Lipinski’s, Veber’s, and Egan’s rules, indicating favorable drug-like properties. The detailed ADME parameters for these compounds are available in Table S3. Furthermore, both compounds exhibited lower toxicity profiles in algae and fish, with rat toxicities classified as class 4, which suggests a lower toxic effect compared with pyrimidine, classified as class 3 or 4. Notably, 2A5BP demonstrated reduced hERG inhibition compared with 2A4CPP and pyrimidine. Additionally, the plasma protein binding for both 2A5BP and 2A4CPP was significantly lower than that observed for pyrimidine, potentially enhancing their bioavailability inside cells and therapeutic efficacy.

### 2.6. Chemical Toxicity in Plant Model and Nematode Models

**Figure 5:**
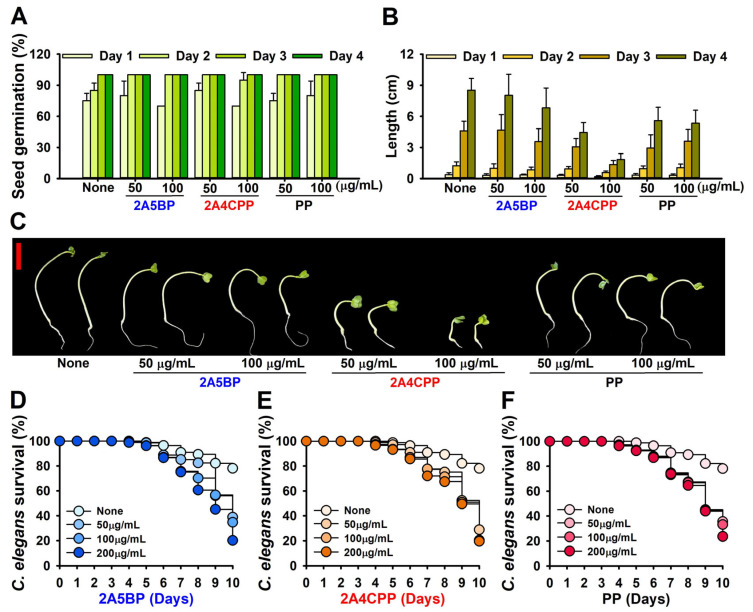
Toxicity of halogenated pyrimidines in the plant and nematode models: Radish seed germination rate (**A**), total length of radish seedlings (**B**), and representative plant images (**C**). cultured with or without different concentrations of 2A5BP, 2A4CPP, and PP at 25 °C. *C. elegans* survival was assessed in the presence or absence of 2A5BP (**D**), 2A4CPP (**E**), and PP (**F**) for 10 days. The red scale bar in (**C**) represents 2 cm.

We conducted toxicity assessments using both plant (radish) and nematode (*C. elegans*) models. The germination rate of radish was unaffected by exposure to 2A5BP and 2A4CPP at concentrations up to 100 μg/mL (Figure 5A). Although plant growth and height remained unchanged in response to 2A5BP and PP, 2A4CPP reduced growth in a concentration-dependent manner (Figure 5B,C). These results suggest that 2A5BP at 100 μg/mL exhibits antibiofilm activity without inhibiting plant growth.

In the *C. elegans* model, all three compounds (2A5BP, 2A4CPP, and PP) showed mild toxic effects on *C. elegans* survival during the 10-day culture period at concentrations up to 200 μg/mL (Figure 5D–F). Overall, these findings emphasize the potential of 2A5BP and 2A4CPP as antibiofilm agents, though their concentration-dependent toxicity necessitates careful dose management for safe application.

## 3. Discussion

This study elucidates the promising antibiofilm and antimicrobial potential of halogenated pyrimidine derivatives against EHEC, a pathogen notorious for its robust biofilm-forming ability and resistance to conventional antibiotics. Notably, compounds such as 2A5BP and 2A4CPP, among the 31 pyrimidine derivatives, have demonstrated significant inhibitory effects on EHEC biofilm formation without adversely affecting bacterial growth, indicating a targeted antibiofilm action rather than a bactericidal effect (Figure 1 and Figure 2).

Colonization of EHEC on epithelial cells results in attaching and effacing lesions in human colons, with Shiga toxin directly contributing to mortality. Currently, appropriate antibiotics are lacking, primarily due to the risk of inducing hemolytic uremic syndrome [20]. The process of EHEC biofilm formation on both biotic and abiotic surfaces is intricate, involving various factors such as cell growth, quorum sensing, motility, curli fimbriae, and other adhesins [21,22]. Particularly, there are numerous small-molecule inhibitors targeting curli production in pathogenic *E. coli* [6,23,24,25,26,27].

The distinction in the mechanisms of action observed between the compounds—where 2A5BP primarily inhibits curli production and 2A4CPP significantly reduces motility and cell growth (Figure 3)—highlights the complex interplay between EHEC’s virulence factors and biofilm development, as previously explored in studies on biofilm mechanisms and control strategies [28]. Notably, qRT-PCR analysis further supports these observations. 2A5BP and 2A4CPP significantly downregulated the expression of key genes involved in curli production, *csgA* and *csgB*, leading to reduced bacterial adhesion and biofilm formation [29]. Specifically, *csgA* encodes the major structural subunit of curli fimbriae, which is crucial for bacterial surface attachment and biofilm development [30]. *csgB*, on the other hand, encodes the nucleator protein essential for initiating the assembly of curli fibers by providing a scaffold for *csgA* incorporation [31]. The downregulation of these genes disrupts the structural integrity of curli fimbriae, thereby significantly impairing the bacteria’s ability to adhere and form biofilms [32].

The mechanism by which inhibition of *csgA* and *csgB* expression reduces biofilm formation is well established [26,33,34,35]. Furthermore, the finding that 2A5BP and 2A4CPP can reduce biofilm integrity without completely inhibiting bacterial growth could represent a strategic advantage in minimizing the selection pressure for resistance development, a critical concern with traditional antimicrobials.

We initially screened pyrimidine, pyrrolo[2,3-d]pyrimidine, and their halogenated derivatives featuring single bromide, chloride, and iodine atoms, as well as two and three halogen atoms, including derivatives with an amine group at the C2 and C4 positions (Table S1). Notably, the presence of an amine group at the C2 position, as seen in 2-amino-5-bromopyrimidine (2A5BP) and 2-amino-4-chloropyrrolo[2,3-d]pyrimidine (2A4CPP), significantly enhanced antibiofilm activity. In contrast, an amine group at the C4 position in compounds such as 5-iodo-7H-pyrrolo[2,3-d]pyrimidin-4-amine (#16) and 4-amino-7H-pyrrolo[2,3-d]pyrimidine (#20) did not exhibit substantial antibiofilm effects (Figure 1 and Table S1). Furthermore, the substitution of a single halogen atom (Br, Cl, I) at the C2 position did not lead to antibiofilm activity, whereas three halogen atoms in the structure of 2,4-dichloro-5-iodo-7H-pyrrolo[2,3-d]pyrimidine (#31) demonstrated significant antibiofilm activity. These observations suggest that the placement of amine or halogen substitutions at the C2 position is crucial for antibiofilm activity. While speculative, varying the substituent groups at the C2 position and increasing halogenation could potentially improve the antibiofilm activity.

Most recently, multiple halogenated pyrimidines have been reported to exhibit antibiofilm activity against *S. aureus* [14] and *V. parahaemolyticus* [15]. In contrast, this study focused on amino-substituted pyrimidines, which showed significant antibiofilm activity against EHEC. These findings highlight the impact of structural differences between halogenated and amino-substituted pyrimidines on antibiofilm efficacy. The differences in antibiofilm effects across species largely depend on the specific characteristics of bacterial strains and the variations in biofilm formation mechanisms, as well as the experimental conditions (e.g., the presence or absence of salts). For example, in *S. aureus*, the halogenated pyrimidine compound 2,4-dichloro-5-fluoropyrimidine effectively inhibited the expression of key biofilm- and virulence-related genes, *agrA* and *hla*, which significantly reduced hemolysis activity [14]. In contrast, *V. parahaemolyticus*, a Gram-negative bacterium, relies on mechanisms like the ToxR system for biofilm formation and maintaining pathogenicity [15]. These differences provide valuable insights for designing chemical modifications tailored to effectively target specific pathogens.

The MIC values observed in this study (400 and 600 µg/mL of 2A5BP and 2A4CPP, respectively) are relatively high, suggesting limited planktonic antimicrobial activity of the tested halogenated pyrimidine compounds. However, the primary focus of this study was not on achieving the lowest possible MIC values but rather on evaluating the antibiofilm efficacy of these compounds while minimizing cytotoxicity. This approach reflects the inherent challenges of addressing biofilm-associated infections, as biofilms are more resistant to conventional antimicrobial agents than planktonic bacteria. We aimed to balance effective biofilm inhibition with low toxicity, ensuring that these compounds could be potentially utilized in therapeutic applications. For example, these compounds effectively inhibit biofilm formation, enabling them to act as potent adjuvants that enhance the efficiency of other antimicrobials in eradicating bacterial cells. In particular, biofilm disruption facilitates easier access for conventional antimicrobials or host immune systems to target bacteria, thereby maximizing overall therapeutic outcomes [36,37].

From an application perspective, the results of this study are particularly relevant for improving sanitation and safety protocols in food processing environments, where EHEC contamination is a significant risk [38]. The deployment of these compounds could be envisioned as part of cleaning regimens or as coatings to prevent biofilm formation on surfaces frequently in contact with food products.

It is important that new antibiofilm agents are less detrimental to human, animal, and ecosystem health. Chemical toxicity assessments using plant and nematode models demonstrated mild toxicity for two aminopyrimidine derivatives, 2A5BP and 2A4CPP (Figure 5). Additional toxicity evaluations with human cell lines or mouse models are required to confirm their safety. Recently, many pyrimidine-based compounds have exhibited low chemical toxicity, indicating their environmental safety [39,40]. Also, the insights gained from the in silico ADME–Tox profiles, which suggest favorable drug-like properties and lower toxicity (Table S3), further support the potential for these compounds to be developed into safe and effective antibiofilm agents. These findings may pave the way for future studies to explore the clinical applicability of these compounds, particularly in formulations that could be used directly in food safety applications or as treatments for established biofilms in medical settings.

## 4. Materials and Methods

### 4.1. Chemicals, Strain, and Growth Conditions

Pyrimidine and thirty-one halogenated pyrimidine derivatives (purity ≥ 98%) were sourced from Combi Blocks (San Diego, CA, USA), as listed in Table S1. These compounds were dissolved in dimethyl sulfoxide (DMSO), purchased from Sigma-Aldrich. For control experiments, 0.1% *v*/*v* DMSO was utilized, which demonstrated no significant effect on the growth or biofilm formation of EHEC at this concentration.

The EHEC strain employed in this study was *Escherichia coli* O157:H7 (EDL933 strain, ATCC 43895). Experimental procedures were performed in Luria–Bertani (LB) medium at 37 °C. To prepare cultures, EHEC cells stored at −80 °C in glycerol stock were first streaked onto LB agar plates and incubated for two days. A fresh single colony was then selected from the agar plate and inoculated into LB broth. For most phenotypic assays, cells cultured for 14–16 h were re-inoculated in LB broth to achieve an optical density of 0.05 at 600 nm (~10^7^ CFU/mL).

### 4.2. Minimum Inhibitory Concentrations (MICs)

To assess the impact of halogenated pyrimidine derivatives on planktonic cell growth under static conditions, approximately 10^7^ CFU/mL of EHEC cells were inoculated into 96-well polystyrene plates (SPL Life Sciences, Pocheon, Republic of Korea) containing LB medium without shaking. The optical density (OD) at 600 nm was measured at 24 h intervals using a Multiskan EX microplate photometer (Thermo Fisher Scientific, Waltham, MA, USA). The MIC was defined as the lowest concentration of pyrimidine derivatives that visibly inhibited the growth of planktonic cells after 24 h. Each assay was performed with at least two independent cultures, with duplicate measurements per culture to ensure reproducibility.

### 4.3. Biofilm Assay

Biofilm formation was assessed using 96-well polystyrene plates (SPL Life Sciences) and quantified via crystal violet staining, following the methodology described [6]. Initially, overnight cultures of EHEC (approximately 10^7^ CFU/mL) were inoculated into LB medium (300 μL) in each well. These cultures were incubated with or without halogenated pyrimidine derivatives at 37 °C for 24 h without agitation. Subsequently, the wells were washed three times with distilled water to remove non-adherent planktonic cells. The biofilms that remained adhered to the wells were stained with 0.1% crystal violet for 20 min, followed by three washes with water to remove excess stain. The bound dye was solubilized with 95% ethanol, and the absorbance of this solution, indicative of the biofilm biomass, was measured at 570 nm using a Multiskan EX microplate photometer (Thermo Fisher Scientific). Results are presented as the mean of at least six independent replicates, conducted using two independent cultures with triplicate measurements per culture.

### 4.4. SEM Analysis of EHEC Biofilms

The effects of halogenated pyrimidine derivatives on the structure of EHEC biofilms were investigated using a field emission-scanning electron microscope (FE-SEM) [6]. Biofilms were cultivated on 4 mm × 4 mm nitrocellulose membranes placed in 96-well plates, incubated with 0, 20, 50, or 100 µg/mL of 2A5BP and 2A4CPP at 37 °C for 24 h. Following incubation, membranes bearing adherent cells were fixed with a solution of 2.5% glutaraldehyde and 2.5% formaldehyde for 24 h at 4 °C. The samples were then sequentially dehydrated in an ethanol series comprising 50%, 70%, 80%, 90%, 95%, and 99% concentrations. After critical-point drying using an HCP-2 apparatus (Hitachi, Tokyo, Japan), the samples were sputter-coated with a palladium/gold alloy and examined under a field emission-scanning electron microscope (FE-SEM, S-4800; Hitachi) at an acceleration voltage of 15 kV. Each assay was replicated with at least two independent cultures, with duplicate measurements per culture to ensure consistency.

### 4.5. Motility Assay

Swimming motility of EHEC was assessed using 0.25% agar plates supplemented with 1% tryptone and 0.25% NaCl, while swarming motility was evaluated on LB broth plates supplemented with 0.5% agar and 0.8% glucose, as previously reported [26]. Plates were prepared by incorporating halogenated pyrimidine derivatives at concentrations of 0, 50, or 100 µg/mL. For the motility assay, a fresh EHEC colony was inoculated into 250 mL flasks containing LB broth and grown to an optical density of 1.0 at 600 nm (approximately 10^7^ CFU/mL). Subsequently, aliquots (0.2 µL) of these cultures were carefully spotted onto the center of each agar plate using sterilized micropipette tips. The plates were then incubated at 37 °C for 9 and 13 h for swimming and swarming assays, respectively. Post incubation, the diameters of the resulting halos were measured to assess motility. Each experimental condition was replicated with at least three independent cultures, with triplicate measurements per culture to ensure reproducibility.

### 4.6. Curli Assay

Curli production in EHEC was assessed using Congo red staining, as described previously [6]. EHEC cells were cultured in LB medium supplemented with 0, 50, or 100 µg/mL of 2A5BP and 2A4CPP. Cultures were maintained in 14 mL round-bottom tubes, agitated at 250 rpm, and incubated at 37 °C for 24 h. Following incubation, cells were harvested by centrifugation at 8000× *g* (r = 0.05 m). Curli production was indicated by the presence of a red color in the cell pellets. Three independent cultures were used, with duplicate measurements per culture.

### 4.7. RNA Isolation and qRT-PCR

To evaluate gene expression changes, an adapted version of a previously described transcriptomic method was employed [26]. EHEC cultures were diluted at a 1:100 ratio in LB medium, consisting of 10 mL LB medium with or without 2A5BP or 2A4CPP (100 μg/mL). The cultures were incubated in 25 mL flasks for 4 h under non-shaking conditions, ensuring that the OD_600_ value exceeded 1. After incubation, 700 μL of RNAlater (Thermo Fisher Scientific Baltics UAB, Vilnius, Lithuania) was added per 20 mL of each culture in a flask and mixed thoroughly to stabilize RNA before further processing.

Cells were collected by centrifugation at 10,000 rpm for 1 min. Glass beads (200–300 μm, Sigma-Aldrich, St. Louis, MO, USA) were added to the lysis buffer for cell disruption. The mixture was subjected to repeated cycles of vortexing for 40 s, followed by chilling on ice for 40 s, and repeated three times to ensure complete cell lysis. After lysis, the supernatant was collected by centrifugation at 12,000 rpm for 1 min, and total RNA was extracted using the Qiagen RNeasy MiniKit (Valencia, CA, USA).

Quantitative real-time PCR (qRT-PCR) was conducted using SYBR™ Green qPCR Master Mix (Applied Biosystems, Foster City, CA, USA) and the ABI StepOne Real-Time PCR System (Applied Biosystems). Primer sequences are listed in Table S2. Ct values were obtained, and the 2^−ΔΔCt^ method was used for analyzing relative gene expression, with *rrsG* as the internal control. Data were collected from two independent cultures with four technical replicates per gene.

### 4.8. In Silico ADME–Tox Analysis

The ADME–Tox (absorption, distribution, metabolism, excretion, and toxicity), drug-likeness, and toxicity profiles of pyrimidine and its halogenated derivatives were comprehensively analyzed using various online web servers. These included PreADMET (https://preadmet.qsarhub.com/, accessed on 5 May 2024), Molinspiration (https://www.molinspiration.com/, accessed on 5 May 2024), and GUSAR (http://www.way2drug.com/gusar/, accessed on 5 May 2024). The validation of these chemical properties was conducted according to the bioassay parameters provided by the servers or through relevant literature citations. A detailed summary of the ADME properties for the halogenated pyrimidine derivatives is presented in Table S3.

### 4.9. Toxicity Assays Using Plant and Nematode Models

To assess the chemical toxicity on the germination and growth of radish (*Raphanus sativus*), seeds were rinsed five times with sterile distilled water and then thoroughly dried [41]. Afterward, ten seeds per plate were placed on soft agar in Murashige and Skoog (MS) plates (0.7% agar, 0.86 g/L MS) with or without 2A5BP, 2A4CPP, and PP at concentrations of 50 and 100 µg/mL. The plates were incubated at room temperature (24 °C) for four days. Seed germination rates and total plant lengths were measured. Four independent experiments were conducted for each sample, with triplicate measurements per experiment.

The *C. elegans* strain *fer-15*(*b26*); *fem-1*(*hc17*) was utilized to investigate the toxicity of 2A5BP, 2A4CPP, and PP in the nematode model, following a previously established protocol [26]. In brief, synchronized adult nematodes were washed twice with M9 buffer (3 g/L KH_2_PO_4_, 6 g/L Na_2_HPO_4_, 5 g/L NaCl, 1 mM MgSO_4_) prior to the experiment. About 30 nematodes were added to each well of 96-well plates containing 300 μL of M9 buffer with 2A5BP, 2A4CPP, and PP at concentrations of 0, 50, 100, or 200 μg/mL. The plates were incubated at 25 °C for 10 days without agitation. This procedure was repeated independently three times with triplicate samples. Results are expressed as percentages of surviving nematodes, determined based on their responses to a 30 s exposure to LED light.

### 4.10. Statistical Analysis

Results are presented as means ± standard deviations. The numbers of independent cultures and replicates are detailed previously. Differences between samples and untreated controls were assessed using Student’s *t*-test. Statistical significance was set at *p*-values less than 0.05 and is indicated by asterisks in the figures.

## 5. Conclusions

In conclusion, the exploration of halogenated pyrimidines as antibiofilm agents offers a novel approach to managing EHEC infections, with implications for both food safety and clinical infection control. These findings encourage further investigation into the molecular mechanisms of action, potential resistance pathways, and practical applications of these compounds.

## Data Availability

The data supporting the findings of this study are available within the article and upon request.

## Supplementary Materials

The supporting information can be downloaded at https://www.mdpi.com/article/10.3390/ijms26031386/s1.
