# Supplementary material for: Antibiofilm Activities of Halogenated Pyrimidines Against Enterohemorrhagic Escherichia coli O157:H7
Source: Int J Mol Sci. 2025 Feb 6;26(3):1386. doi: 10.3390/ijms26031386 (PMC11818689; doi:10.3390/ijms26031386)
Supplement: Supplementary file 1 [file ijms-26-01386-s001.zip › ijms-3401958-supplementary.pdf]

International Journal of *Molecular Sciences*

MDPI

Article

Antibiofilm activities of halogenated pyrimidines against enterohemorrhagic *Escherichia coli* O157:H7

Hyejin Jeon [1], Yong-Guy Kim [1], Jin-Hyung Lee [1,*] and Jintae Lee [1,*]

[1] School of Chemical Engineering, Yeungnam University, Gyeongsan, 38541, Republic of Korea; hyejin7882@ynu.ac.kr (H.J.); yongguy7@ynu.ac.kr (Y.-G.K.)

* Correspondence: jinhlee@ynu.ac.kr (J.-H.L.); jtlee@ynu.ac.kr (J.L.); Tel.: +82-53-810-3812 (J.-H.L.); Tel.: +82-53-810-2533 (J.L.)

Citation:

Academic Editor(s): Name

Received: date
Revised: date
Accepted: date
Published: date

Copyright: © 2024 by the authors. Submitted for possible open access publication under the terms and conditions of the Creative Commons Attribution (CC BY) license (https://creativecommons.org/licenses/by/4.0/).

Table S1. Antimicrobial and antibiofilm activities of halogenated pyrimidines. Biofilm formation (expressed as percentages compared to untreated controls) was measured at OD_{570} in the presence of each halogenated indole at 0, 20, or 100 μg/mL after culture for 24 h in 96-well plates without shaking, and planktonic cell growth (also expressed as percentages compared to untreated controls) was simultaneously measured at OD_{600}.

No.	Halogenated pyrimidines	Structures	Biofilm (%)		Cell growth (%)	
			20	100	20	100
1	5-Iodopyrimidine	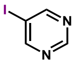	81 ± 18	103 ± 10	122 ± 12	126 ± 7
2	4-Chloro-5-Iodopyrimidine	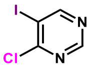	68 ± 17	48 ± 15	103 ± 10	71 ± 2
3	5-Iodo-2,4-dimethoxypyrimidine	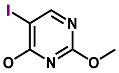	72 ± 17	69 ± 6	112 ± 14	105 ± 5
4	5-Bromopyrimidine	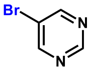	81 ± 19	81 ± 10	116 ± 10	113 ± 16
5	2-Amino-5-bromopyrimidine	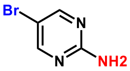	65 ± 11	14 ± 10	106 ± 2	73 ± 3
6	5-Bromo-2-iodopyrimidine	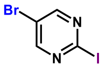	77 ± 21	73 ± 12	106 ± 17	115 ± 10
7	2-Chloropyrimidine	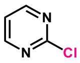	81 ± 24	94 ± 15	117 ± 6	103 ± 8
8	2-Chloro-5-fluoropyrimidine	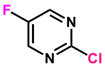	88 ± 21	93 ± 12	112 ± 14	108 ± 14
9	2,4-Dichloro-5-fluoropyrimidine	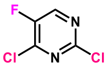	88 ± 5	77 ± 13	98 ± 5	73 ± 7
10	4,6-Difluoropyrimidine	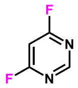	88 ± 6	100 ± 9	96 ± 7	106 ± 5
11	5-Chloro-2-iodopyrimidine	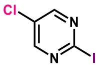	83 ± 4	102 ± 9	92 ± 5	103 ± 8
12	5-Bromo-2-chloropyrimidine	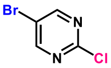	84 ± 6	105 ± 7	96 ± 5	112 ± 7
13	7H-Pyrrolo[2,3-d]pyrimidine	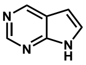	86 ± 4	89 ± 9	101 ± 11	113 ± 8
14	5-Iodo-7H-pyrrolo[2,3-d]pyrimidine	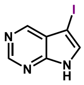	81 ± 12	63 ± 5	89 ± 6	85 ± 9
15	2-Chloro-5-iodo-7h-pyrrolo[2,3-d] pyrimidine	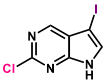	80 ± 5	64 ± 8	84 ± 8	115 ± 7

16	5-Iodo-7h-pyrrolo[2,3-d]pyrimidin-4-amine	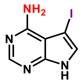	83 ± 6	50 ± 9	96 ± 14	71 ± 2
17	5-Bromo-4-chloro-7h-pyrrolo[2,3-d]pyrimidine	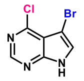	64 ± 8	50 ± 7	107 ± 11	68 ± 2
18	4-Bromo-7H-pyrrolo[2,3-D]pyrimidine	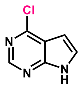	64 ± 10	79 ± 8	115 ± 12	98 ± 3
19	5-Bromo-2-chloro-7H-pyrrolo[2,3-d]pyrimidine	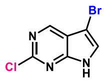	60 ± 8	54 ± 9	121 ± 11	107 ± 7
20	4-Amino-7H-pyrrolo[2,3-d]pyrimidine	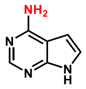	72 ± 9	99 ± 9	118 ± 18	107 ± 10
21	4-Chloro-7H-pyrrolo[2,3-d]pyrimidine	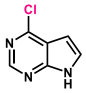	63 ± 8	75 ± 9	115 ± 16	99 ± 10
22	4,5-Dichloro-7H-pyrrolo[2,3-d]pyrimidine	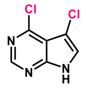	67 ± 11	85 ± 9	113 ± 21	99 ± 7
23	4-Chloro-7H-pyrrolo[2,3-d]pyrimidin-2-amine	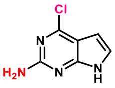	66 ± 13	2 ± 1	96 ± 3	34 ± 5
24	2-Chloro-7h-pyrrolo[2,3-d]pyrimidine	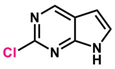	70 ± 11	74 ± 13	115 ± 23	72 ± 4
25	5-Bromo-2,4-dichloro-7h-pyrrolo[2,3-d]pyrimidine	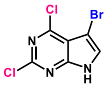	59 ± 9	21 ± 6	94 ± 12	49 ± 4
26	2,4-Dichloro-7H-pyrrolo[2,3-d]pyrimidine	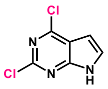	71 ± 16	38 ± 6	103 ± 9	104 ± 3
27	4-Chloro-7-methyl-7H-pyrrolo[2,3-d]pyrimidine	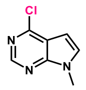	75 ± 11	55 ± 5	120 ± 13	101 ± 9
28	4-Chloro-2-methyl-7h-pyrrolo[2,3-d]pyrimidine	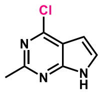	68 ± 10	47 ± 7	107 ± 13	100 ± 13
29	4-Chloro-6-iodo-7h-pyrrolo[2,3-d]pyrimidine	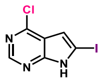	69 ± 11	71 ± 5	130 ± 13	136 ± 11
30	7-Benzyl-4-chloro-7h-pyrrolo[2,3-d]pyrimidine	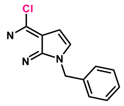	67 ± 12	46 ± 8	112 ± 7	91 ± 13
31	2,4-Dichloro-5-iodo-7H-pyrrolo[2,3-d]pyrimidine	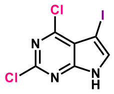	52 ± 11	13 ± 5	90 ± 10	29 ± 1
32	Pyrimidine	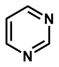	83 ± 15	90 ± 9	117 ± 23	116 ± 12

Table S2. Primers used for RT-PCR

Gene	Functions	Primer
csgA	Curli subunit, major curli subunit	Forward: 5'-AGA TGT TGG TCA GGG CTC AG-3'<br>Reverse: 5'-CGT TGT TAC CAA AGC CAA CC-3'
csgB	Curli nucleator protein curlin CsgB	Forward: 5′-ATC AGG CAG CCA TAA TTG GT-3′<br>Reverse: 5′-CCA TAA GCA CCT TGC GAA AT-3′
flhD	Flagellar transcriptional regulator FlhD; motility	Forward: 5'-TGC ATA CCT CCG AGT TGC TG-3'<br>Reverse: 5'-GCG TGT TGA GAG CAT GAT GC-3'
fliA	RNA polymerase sigma factor for flagellar operon	Forward: 5'-TTA GGG ATC GAT ATT GCC GAT T-3'<br>Reverse: 5'-CGT AGG AGA AGA GCT GGC TGT T-3'
luxR	Quorum sensing	Forward: 5'-GGC CGA ACT CGT AAA ATG G-3'<br>Reverse: 5'-ATT AAG TTT CGC CGG GTG AC-3'
motB	Ciliary or flagellar motility	Forward: 5'-CAG GGG GAA GTG AAT AAG CA-3'<br>Reverse: 5'-TTC TAA ACA TCG GGC GAT TC-3'
stx2	Shiga-like toxin 2	Forward: 5'-GTT CCG GAA TGC AAA TCA GT-3'<br>Reverse: 5'-CGG CGT CAT CGT ATA CAC AG-3'
rrsG	16S ribosomal RNA (Housekeeping gene)	Forward: 5'-TAT TGC ACA ATG GGC GCA AG-3'<br>Reverse: 5'-ACT TAA CAA ACC GCC TGC GT-3'

Table S3. ADME-Tox profiles of pyrimidine, 2A5BP, 2A4CPP, and PP.

Property	Pyrimidine	2A5BP	2A4CPP	PP
Lipinski rule of five	Suitable	Suitable	Suitable	Suitable
Lipinski rule of five violations	0	0	0	0
Lipinski	Yes; 0 violation	Yes; 0 violation	Yes; 0 violation	Yes; 0 violation
Veber	Yes	Yes	Yes	Yes
Egan	Yes	Yes	Yes	Yes
Algae at	0.3811	0.1648	0.1168	0.2725
Acute fish toxicity (medaka)	19.7529	1.7354	0.3032	3.6547
Acute fish toxicity (minnow)	2.2805	0.8091	0.1786	0.7865
Rat oral LD50 Classification	Class 4 in AD	Class 4 in AD	Class 4 in AD	Class 3 in AD
Rat IP LD50 Classification	Class 4 in AD	Class 4 in AD	Class 4 in AD	Class 4 in AD
Rat IV LD50 Classification	Class 3 in AD	Class 4 in AD	Class 4 in AD	Class 4 in AD
Rat SC LD50 Classification	Class 3 in AD	Class 4 in AD	Class 4 in AD	Class 4 in AD
hERG inhibition	Medium risk	Low risk	Medium risk	Medium risk
Plasma Protein Binding	62.38	1.75	7.22	4.35
Bioavailability Score	0.55	0.55	0.55	0.55
Rat carcinogenicity	Negative	Positive	Negative	Negative
Mouse carcinogenicity	Negative	Negative	Positive	Negative
TPSA	25.78	51.81	67.60	41.58
GPCR ligand	-3.47	-2.93	0.07	-1.27

Ion channel modulator	-3.28	-2.90	0.31	-0.55
Kinase inhibitor	-3.11	-2.62	0.50	0.01
Nuclear receptor ligand	-3.74	-3.67	-1.77	-3.03
Protease inhibitor	-3.64	-3.38	-1.00	-1.86
Daphnia at	4.8079	1.2122	0.4622	1.8230
Ghose	No; 3 violations: MW<160, MR<40, #atoms<20	No; 2 violations: MR<40, #atoms<20	No; 1 violation: #atoms<20	No; 3 violations: MW<160, MR<40, #atoms<20
Muegge	No; 2 violations: MW<200, #C<5	No; 2 violations: MW<200, #C<5	No; 1 violation: MW<200	No; 1 violation: MW<200
GI absorption	High	High	High	High
BBB permeant	No	Yes	No	Yes
P-gp substrate	No	No	No	No
CYP1A2 inhibitor	No	No	No	No
CYP2C19 inhibitor	No	No	No	No
CYP2C9 inhibitor	No	No	No	No
CYP2D6 inhibitor	No	No	No	No
CYP3A4 inhibitor	No	No	No	No
Leadlikeness	No; 1 violation: MW<250	No; 1 violation: MW<250	No; 1 violation: MW<250	No; 1 violation: MW<250
Enzyme inhibitor	-3.31	-2.35	0.59	-0.71

ADME: absorption, distribution, metabolism, excretion, hERG: Human ether-a-go-go related gene, TPSA: Topological polar surface area, GPCR: G protein-coupled receptor, IP: Intraperitoneal route of administration, IV: Intravenous route of administration, SC: Subcutaneous route of administration, in AD: compound falls in applicability domain of models.
